# The p48 Flow Modulation Device with Hydrophilic Polymer Coating (HPC) for the Treatment of Acutely Ruptured Aneurysms: Early Clinical Experience Using Single Antiplatelet Therapy

**DOI:** 10.1007/s00270-020-02418-4

**Published:** 2020-02-06

**Authors:** Marta Aguilar-Perez, Victoria Hellstern, Muhammad AlMatter, Christina Wendl, Hansjörg Bäzner, Oliver Ganslandt, Hans Henkes

**Affiliations:** 1 Neuroradiologische Klinik, Neurozentrum, Klinikum Stuttgart, Stuttgart, Germany; 2 Institut für Röntgendiagnostik, Zentrum für Neuroradiologie, Universitätsklinikum Regensburg, Regensburg, Germany; 3 Neurologische Klinik, Neurozentrum, Klinikum Stuttgart, Stuttgart, Germany; 4 Neurochirurgische Klinik, Neurozentrum, Klinikum Stuttgart, Stuttgart, Germany; 5 Medizinische Fakultät, Universität Duisburg-Essen, Essen, Germany; 6 Present Address: Neuroradiologische Klinik, Klinikum Stuttgart, Kriegsbergstrasse 60, 70174 Stuttgart, Germany

**Keywords:** Ruptured aneurysm, Blister-like aneurysm, Dissecting aneurysm, Aneurysmal subarachnoid hemorrhage, Flow diverter, Surface modification, HPC, p48MW HPC, Single antiplatelet therapy

## Abstract

**Background:**

Flow diversion (FD) remains a potential treatment option following aneurysmal subarachnoid hemorrhage (aSAH) when standard options may not be feasible. However, it should not be considered a first-line treatment due to the need for dual antiplatelet therapy (DAPT). The hydrophilic polymer coating on the p48MW flow diverter (HPC, phenox) is a surface modification that inhibits platelet adhesion. This study aims to report on our early single-center experience using the p48MW HPC (phenox) flow diverter with single antiplatelet therapy (SAPT) following an aSAH.

**Materials and Methods:**

We retrospectively identified all patients who had been treated with the p48MW HPC for aSAH under SAPT. All patients treated within 30 days following an aSAH were included. Any occurrence of thromboembolic and hemorrhagic complications was recorded alongside angiographic and clinical follow-up details.

**Results:**

Eight patients were identified. The mean interval between aSAH and FD was 6 days. Of the eight ruptured aneurysms, one was blister-like, one saccular, one mycotic, and the remaining five were dissecting aneurysms. Intraprocedural transient thrombus formation was observed in four patients (50%). Stent thrombosis was observed in one patient (12.5%) on day 3 with spontaneous recanalization after being switched onto DAPT. None of the aneurysms rebled after treatment. Two patients died due to cerebral vasospasm. Complete aneurysm occlusion had been achieved in all but one patient at angiographic follow-up (average 6 months).

**Conclusions:**

This small series highlights the possibility and limitations of using the p48MW HPC with SAPT in ruptured aneurysms. Randomized trials with longer follow-up in larger cohorts are underway.

## Introduction

The rationale behind an early intervention following an aneurysmal subarachnoid hemorrhage (aSAH) is to prevent rebleeding. Endovascular coiling and surgical clipping are the two primary treatment modalities used for acutely ruptured aneurysms. However, each of these treatment modalities has limitations [1]. Flow diversion (FD) has emerged as a treatment option for unruptured aneurysms due to its high angiographic occlusion rates [2]. However, the use of flow diverter stents (FDS) in the presence of an aSAH is limited due to the need for dual antiplatelet therapy (DAPT) and the delay in the aneurysm occluding, which is potentially associated with hemorrhagic complications.

A variety of different FDS are currently available on the market. The p48MW (phenox, Bochum, Germany) is a new FDS that has been designed to treat distal aneurysms in vessels with diameters of between 1.75 and 3 mm [3]. Its hydrophilic polymer coating (pHPC, phenox, Bochum, Germany) is a novel glycan-based multilayer polymer that has been shown to have significant anti-thrombogenic properties when tested in vitro [4]. A recently published study concluded that the p48MW HPC appears both safe and efficacious under single antiplatelet therapy (SAPT) using prasugrel in selected cases of unruptured aneurysms [5]. However, no publications have yet focused on the use of this surface-modified FDS in the setting of aSAH. In this retrospective study, we present our early experience in using the p48MW HPC under SAPT for ruptured aneurysms.

## Materials and Methods

### Device Description

The p48MW HPC comprises 48 braided drawn solid tubular wires. Each strand is made of platinum-filled nitinol tubing and coated with a recently developed glycan-based multilayer hydrophilic polymer coating (pHPC, phenox). Unlike the p64 flow modulation device (phenox), the p48MW HPC is not mechanically detached. A proximal radio-opaque marker on the insertion wire identifies the point at which the device can still be re-sheathed. There is a central, independently moveable stainless steel wire with an atraumatic distal nitinol wire tip to prevent small distal vessels from rupturing. The device is compatible with 0.021″ inner diameter microcatheters and is currently available with a nominal diameter of 2 mm and 3 mm, designed to treat vessels of between 1.75 and 3 mm diameter, respectively.

### Patient Selection

We retrospectively reviewed our prospectively maintained database to identify all patients treated with a p48MW HPC under SAPT within 30 days of an aSAH. We also included cases in which FD was performed within 30 days of hemorrhage, but in a separate intervention after the initial procedure. Patients who underwent staggered FD of an aneurysmal remnant after 30 days were, however, excluded.

For each patient, we recorded demographic data, clinical presentation, morphology and location of the ruptured aneurysm, size and location of the implanted p48MW HPC, procedural and general complications, and the clinical and radiological outcomes.

### Endovascular Treatment and Antiplatelet Regimen

All treatments were performed under general anesthesia using biplane digital subtraction angiography (DSA) systems (Axiom, Siemens, Erlangen, Germany) and using either a 6F or an 8F right femoral artery approach. The treatment strategy used was jointly decided by a senior neurointerventionist and a senior vascular neurosurgeon. Depending on the timing of the procedure, patients received either 1 × 100 mg acetylsalicylic acid (ASA, Aspirin, Bayer Vital, Leverkusen, Germany) or 1 × 10 mg prasugrel (Efient, Daiichi Sankyo, Munich, Germany) PO daily for at least 3 days before treatment, or a loading dose of 1 × 500 mg ASA (Aspirin IV 500 mg, Bayer Vital) IV or 1 × 30 mg prasugrel PO 3 h before the procedure. The effectiveness of the antiplatelet regimen was tested in all cases before treatment using the Multiplate Analyzer (Roche Diagnostics, Mannheim, Germany) and VerifyNow (Accriva, San Diego, CA, USA). The maintenance dose was monitored by repeated testing, starting with standard daily doses of 1 × 500 mg ASA IV or 1 × 10 mg prasugrel PO. Patients were temporarily heparinized during the procedure (typically with a bolus of 3000 IU unfractionated heparin IV). Additionally, all flushing solutions, including the guiding catheters and microcatheters, were heparinized (5000 IU unfractionated heparin/l). If thrombus formation occured during the procedure, a body weight-adapted bolus of 180 mcg/kg eptifibatide IV (Integrilin, GlaxoSmithKline, Munich, Germany) was administered. If there proved to be insufficient platelet inhibition under SAPT (confirmed by daily response testing using Multiplate and VerifyNow) *with* thrombus formation during the postprocedural period, DAPT was started. Should, however, there have been insufficient inhibition under SAPT *without* apparent thrombus formation, the maintenance dose was increased, but no DAPT was initiated.

### Data Collection and Follow-Up

Patency and flow characteristics within the parent vessel were assessed angiographically immediately after the FDs were placed. Follow-up angiographies by DSA combined with clinical evaluation were performed during the first week after treatment and again at 3–6 months. Aneurysm occlusion was graded using the Raymond–Roy classification [6].

## Results

We identified eight patients (three women and five men) who were treated within 30 days of an aSAH using one or more p48MW HPC stents. The mean age at presentation was 60 years (range 49–73). The decision to use a FDS was decided upon as a part of the primary treatment strategy in 7 (87.5%) of the cases included, while in the remaining case (12.5%), it was a secondary decision once partial primary coiling had been carried out. The clinical condition upon presentation was excellent (Hunt and Hess (HH) I–II), moderate (HH III), and poor (HH IV–V) in 1 (12.5%), 2 (25%), and 5 (62.5%) patients, respectively. The ruptured aneurysms comprised one saccular aneurysm of the anterior communicating artery (AcomA), one mycotic aneurysm of the right middle cerebral artery (MCA) M2 segment, one blister-like aneurysm of the basilar artery (BA), one dissecting aneurysm of the BA, two dissecting aneurysms of the intradural left internal carotid artery (ICA), and two dissecting aneurysms of vertebral artery (VA) V4 segments. Table 1 summarizes the baseline demographics, aneurysms characteristics, and antiplatelet therapy in this series.

**Table 1:** Baseline demographics, aneurysm characteristics, and antiplatelet therapy

Patient No.	Gender	Age (years)	Location	Morphology	Fisher Grade	Hunt and Hess Grade	APT prior treatment	Intraprocedural heparin (IV bolus)	Final daily APT after treatment with daily test monitoring
1	F	73	ICA (left)	Dissecting	IV	II	1500 mg ASA IV	5000 IU	2 × 500 mg ASA IV
2	M	62	AcomA	Saccular	IV	III	1 × 100 mg ASA PO for 2 weeks	3000 IU	1 × 100 mg ASS PO + 2 × 90 mg Ticagrelor PO
3	M	66	BA	Blister	IV	III	1 × 10 mg Prasugrel PO for 3 days	3000 IU	2 × 10 mg Prasugrel PO
4	M	67	V4 (left)	Dissecting	IV	V	1000 mg ASA IV	–	2 × 500 mg ASA IV
5	M	57	V4 (right)	Dissecting	IV	V	1000 mg ASA IV	3000 IU	2 × 500 mg ASA IV + 2 × 180 mg Ticagrelor PO
6	M	50	MCA (right)	Mycotic	IV	IV	1500 mg ASA IV	3000 IU	3 × 500 mg ASA IV
7	F	58	BA	Dissecting	IV	IV	1000 mg ASA IV	–	3 × 500 mg ASA PO + 2 × 180 mg Ticagrelor PO
8	F	49	ICA (left)	Dissecting	III	IV	30 mg Prasugrel PO	–	1 × 10 mg Prasugrel PO

Two patients (patient #1 and #6) presented with a second aSAH shortly after a previous aSAH from an independent aneurysm. Patient #1 suffered an aSAH from a ruptured left MCA aneurysm that was surgically clipped. The neurosurgeon encountered intraoperative bleeding from the ICA, but the possible origin was not visible. Fourteen days later, the patient suffered a second aSAH from a de novo dissecting left supraclinoid ICA aneurysm, treated with a p48MW HPC. Patient #6 had a previous history of long-term drug- and alcohol consumption, and during acute endocarditis, he suffered an aSAH from a left MCA M2 mycotic aneurysm that was treated with endovascular parent vessel occlusion. Four days later, the patient suffered a second aSAH from a de novo right MCA M2 mycotic aneurysm, which was treated with a p48MW HPC.

**Fig. 1:**
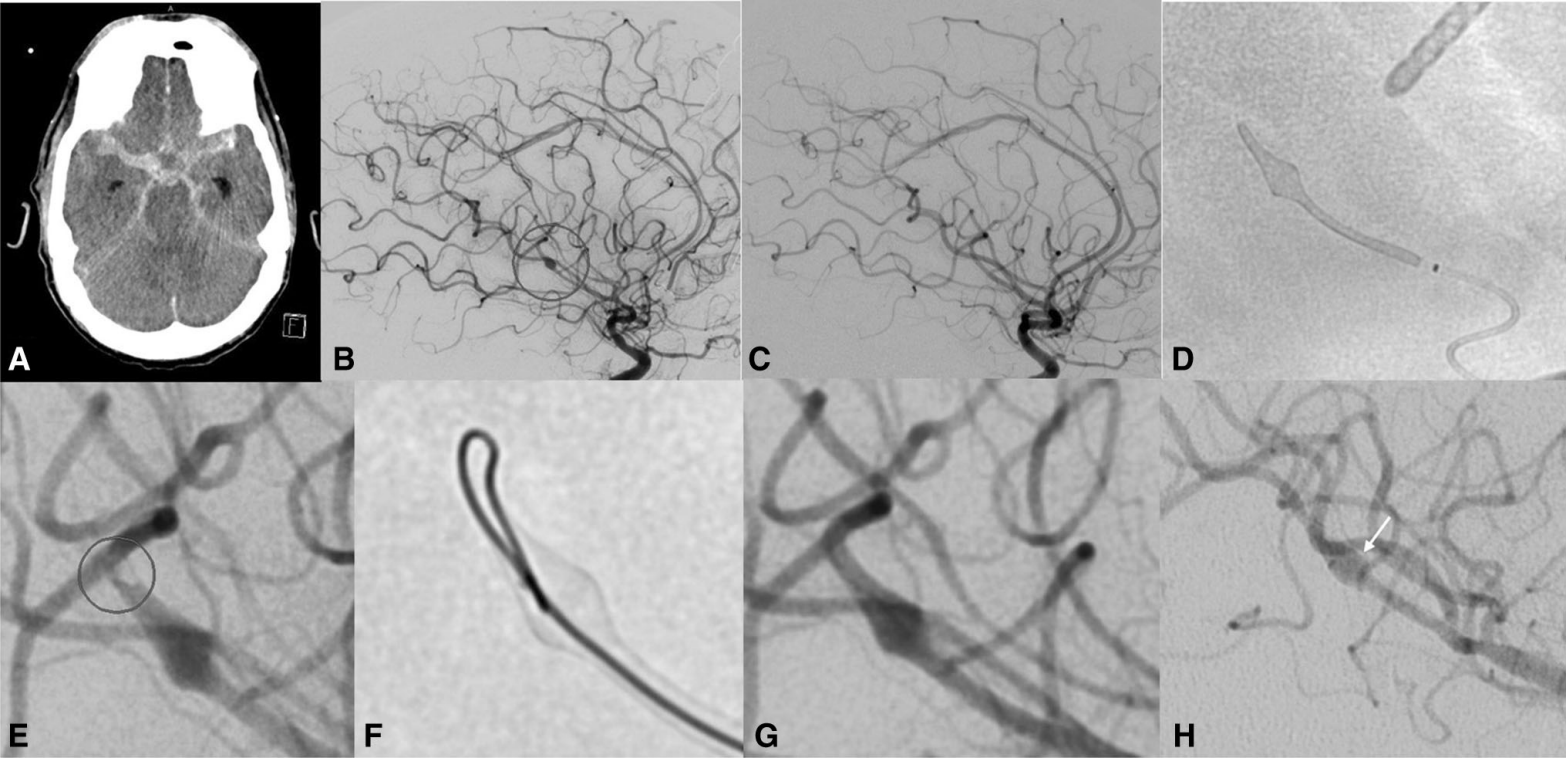
A 50-year-old and drug-addicted male presented with subarachnoid hemorrhage from a left mycotic MCA aneurysm, who was treated by parent vessel occlusion (Patient #6). Four days later, and with the patient still intubated, he presented a sudden increase in the intracranial pressure. Head CT scan revealed a second subarachnoid hemorrhage, more prominent on the right side (**A**). A new diagnostic angiography showed a de novo right M2 mycotic aneurysm (**B**, circle), which was not present in the initial angiography (**C**). A single p48MW HPC (3 mm × 18 mm) was implanted after premedication with ASA (**D**). A total of 1500 mg ASA IV was needed to achieve sufficient platelet inhibition, tested by Multiplate and VerifyNow. After deployment, an incomplete opening of the distal end of the p48MW HPC with small thrombus formation was observed (**E**, circle). The complete opening of the device was achieved after crossing with an inverted microguidewire (**F**). The thrombus formation resolved after injection of eptifibatide (**G**). The most recent follow-up angiography performed 8 months after treatment showed the p48MW HPC following the contour of the fusiform vessel enlargement (**H**, arrow)

An external ventricular drain (EVD) was needed for seven patients (87.5%). In each case, the EVD was inserted before initiating the antiplatelet medication. No hemorrhage along the EVD track was noticed on imaging before or after endovascular treatment. The FDS was deployed at a median latency of 6 days (range 2-26) after the hemorrhage. A single FDS was used in five patients (62.5%), two devices in two patients (25%), and multiple devices in one patient (12.5%). Delivery and deployment of the FDS were straightforward in all but two cases (25%). Patient #2 presented some vasospasm immediately distal to the p48MW HPC after the device had to be repositioned a single time to obtain a satisfactory position. In patient #6, the distal end of the p48MW HPC was seen to have incompletely opened, an issue which resolved after being crossed with an inverted microguidewire (Fig. 1).

### Antiplatelet Therapy

SAPT with ASA was used for six patients (75%), while for the remaining two patients, prasugrel was used as SAPT (25%). The maintenance doses were monitored by repeated testing. All but one patient (83.3%) receiving ASA required increased dosages of ASA before and after treatment to achieve sufficient platelet inhibition. The patients who received prasugrel showed only minor variability in their platelet inhibition before and after the procedure.

DAPT was started in the postoperative period in three patients (37.5%), all of them receiving ASA as their SAPT regime. In patient #2, DAPT was started due to the small diameter of the treated vessel (A1/A2) and the presence of some vasospasm, which may have increased the risk of in-stent thrombosis. In patient #5, DAPT was started due to delayed thrombus formation secondary to vasospasm and reduced platelet inhibition under ASA, and in patient #7 due to in-stent thrombosis on day 3 after treatment. No patient under prasugrel had to be switched to DAPT.

### Complications and Early Angiographic Results

Intraprocedural thrombus formation inside or in the vicinity of the FDS was observed in a total of 4 patients (50%). However, in patients #2 and #6, this thrombus formation was probably not directly related to the surface of the implanted device, rather induced by vessel vasospasm and the device incompletely opening, respectively. In patients #1 and #5, thrombus formation was observed at the origin of the left A1 segment and the right posterior-inferior cerebellar artery (PICA), respectively. Since the FDS jailed the origins of both vessels, we assume that the thrombus formation was related to reduced flow in those FDS-covered vessels rather than to the device itself. All thrombi resolved once a bolus of eptifibatide IV had been administered. There were no clinical or radiological sequelae of these thrombus formations. Patient #3 and #7 presented with pontine ischemia after the FDS had been deployed into the BA (25%). In both patients, pontine branches originated adjacent to BA aneurysms and occluded alongside the obliterated aneurysm. No other ischemic lesions directly related to the FDS were observed.

**Fig. 2:**
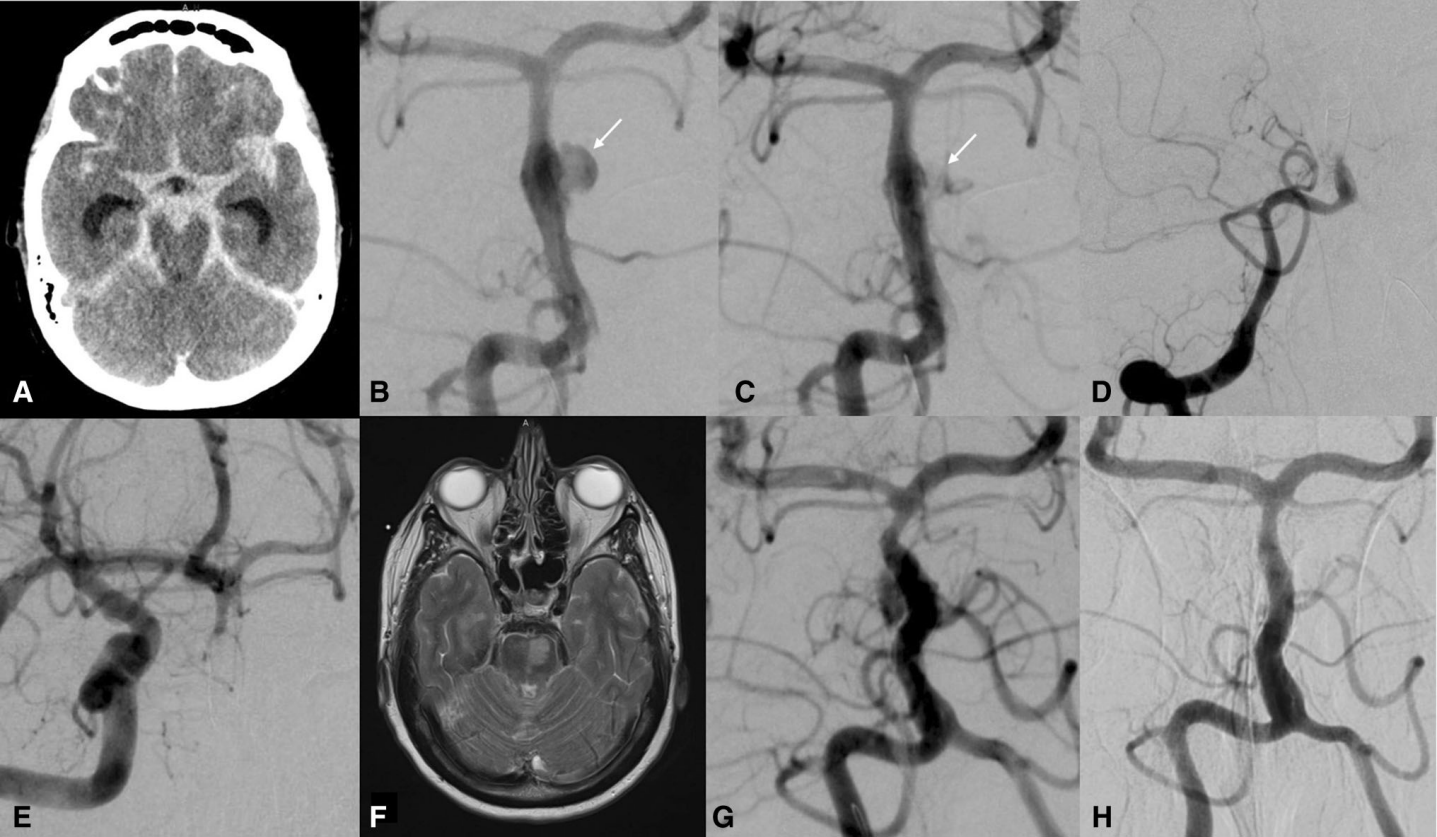
A 58-year-old female presented with a sudden headache after impact with the trunk lid, followed by deterioration with nausea, vomiting, and unconsciousness (Patient #7). A cranial CT scan obtained on the day of admission revealed diffuse subarachnoid hemorrhage (Fisher grade 4) and hydrocephalus (**A**). Posterior–anterior arteriogram obtained with a right vertebral artery injection showing a fusiform dissecting aneurysm of the BA with the formation of a pseudoaneurysm (arrow) (**B**). Endovascular treatment was performed on day 1 after bleeding. Multiplate and VerifyNow tests confirmed sufficient platelet inhibition after premedication with 1000 mg ASA IV. Posterior–anterior projection obtained immediately after telescopic deployment of 5 p48MW HPC FDS (**C**). Insufficient platelet inhibition by ASA after treatment resulted in in-stent thrombosis on day 3 (**D**). The distal part of the BA was retrogradely perfused via the posterior communicating artery (**E**). T2WI MRI is showing a left pontine infarction due to the thrombosis of a pontine branch together with the dissecting aneurysm (**F**). Recanalization of the BA, including the aneurysm, was observed after the switch to DAPT plus eptifibatide IV (**G**). The patient was retreated with two p48MW HPC implanted distally before discharge. The most recent follow-up angiography performed 9 months after treatment shows complete exclusion of the pseudoaneurysm with total reconstruction of the previously dissected BA and patency of the FDS (**H**)

Early angiographic follow-up was performed during the first week after treatment in all patients (median 5 days). It showed complete aneurysm occlusion in three patients (37.5%), reconstruction of the parent vessel with minor perfusion of the aneurysm in three patients (37.5%), and residual aneurysm filling in two patients (25%). At that time, non-occlusive thrombus formation was observed inside the FDS in two patients (patient #1 and #5 (25%)). Patient #1 presented with insufficient platelet function inhibition under ASA, confirmed by Multiplate and VerifyNow. Patient #5 presented with massive cerebral vasospasm of the right V4 segment, which resulted in a functional occlusion of the FDS. Blood flow stagnation, together with an insufficient platelet function inhibition under ASA, resulted in a thrombus formation inside the FDS, which ultimately resolved after the switch to DAPT and aggressive vasospasm therapy. Whether this thrombus formation influenced the clinical outcome of these two patients remains unclear since both were still intubated before and after the DSA. At the least, CT and MRI did not reveal cerebral ischemia as a result of these events. In-stent thrombosis was observed in patient #7 on day 3 after the treatment (12.5%), which was also related to insufficient platelet function inhibition under ASA, confirmed by response tests. The distal part of the BA was retrogradely perfused via the posterior communicating artery (PcomA). Recanalization of the FDS inside the BA was observed after the switch to DAPT plus eptifibatide IV (Fig. 2). Under this medication, the patient presented gastrointestinal bleeding that required embolization. No other major hemorrhagic complications were observed in this series.

### Clinical and Angiographic Follow-up

**Fig. 3:**
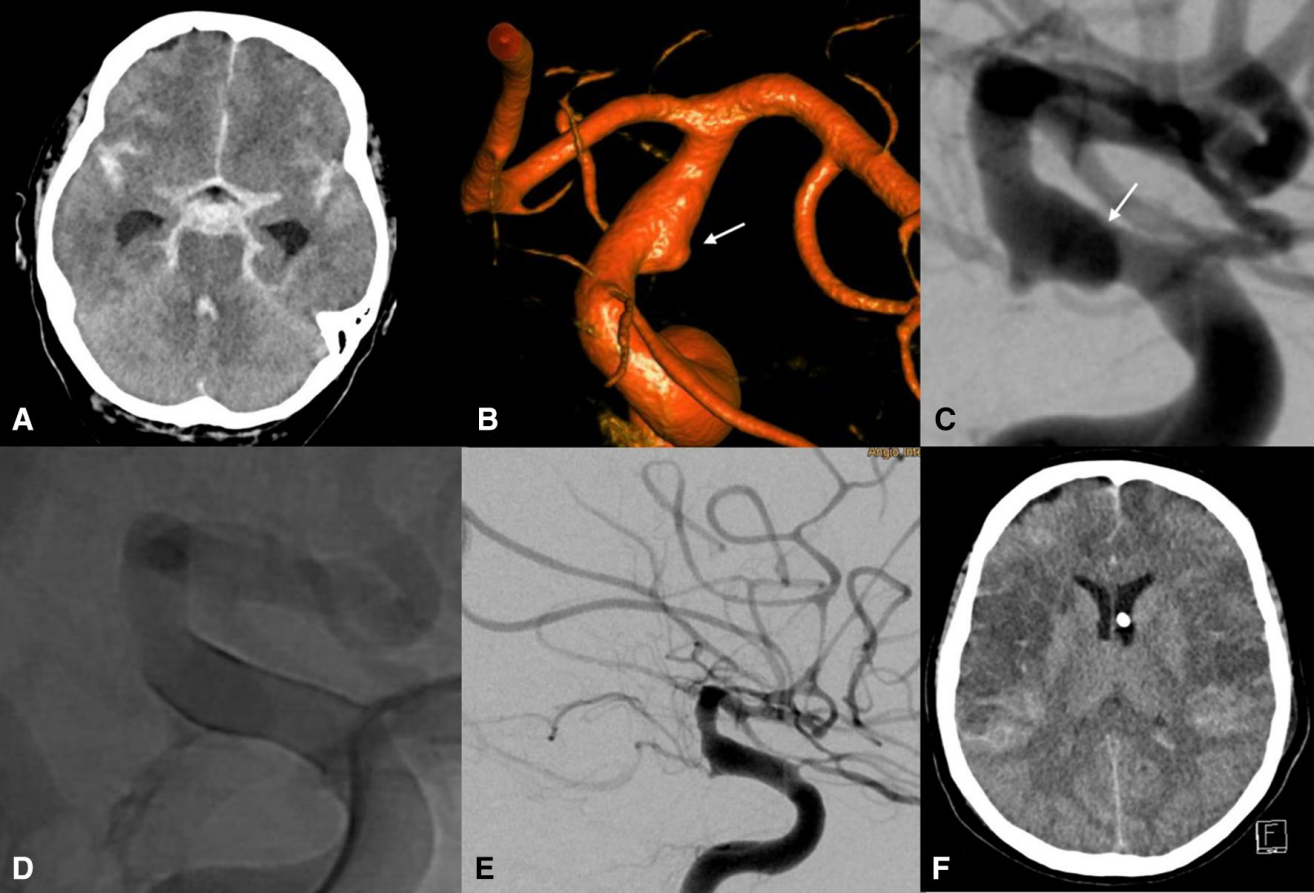
A 49-year-old female presented with sudden onset of severe headache and subsequent obtundation (Patient #8). Cranial CT scan (**A**) showed diffuse subarachnoid hemorrhage (Fisher grade 4) and hydrocephalus. A diagnostic cerebral angiography (**B**, 3D-Volumen Rendering Technique; **C**, lateral view) demonstrated a fusiform, most likely dissecting left ICA paraclinoid aneurysm with a non-discernible neck (**B**, **C**, arrow). The aneurysm was treated with a single p48MW HPC (3 mm × 18 mm) after premedication with 30 mg prasugrel PO 3 h before the procedure (**D**). Multiplate and VerifyNow test confirmed sufficient platelet inhibition before and after treatment. Angiographic follow-up performed on day 2 after treatment showed the patency of the FDS and intracranial massive cerebral vasospasm (**E**). Refractory cerebral vasospasm resulted in large bilateral MCA and ACA infarction (**F**) and eventually, death

No rebleeding from the treated aneurysms occurred. Two patients died due to refractory cerebral vasospasm during the postoperative period (Fig. 3). Midterm angiographic follow-up was available for the remaining six patients at an average of 6 months postoperatively (range 3-9 months). In five patients (83%), the aneurysm was completely occluded. In patient #6, the fusiform aneurysm remained unchanged after 8 months with the implanted p48 HPC following the contour of the fusiform vessel enlargement. The infectious origin of said aneurysm was considered to be a possible explanation for this phenomenon.

All patients available for angiographic follow-up were also clinically evaluated based on mRS. Three patients showed good functional recovery after the SAH with mRS ≤ 2 (50%), and three patients presented at mRS > 2 (50%; one patient with mRS 5, and two patients with mRS 4). Table 2 summarizes the clinical and angiographic follow-up results in this series.

**Table 2:** Clinical and radiographic outcome

Patient No.	p48 HPC size	Position of the FDS	Time between aSAH and treatment (days)	Additional coils	Intraprocedural complication	Postprocedural complication related with the treatment	Retreatment	Occlusion at delayed FU (months)	mRS at delayed FU
1	3/12 mm	M1/ICA (left)	0	No	Thrombus formation	None	None	–	6
2	3/12 mm	A2/A1 (right)	26	Yes	Vasospasm (distal) Thrombus formation	None	2/15 mm (A2/A1 left)	Total occlusion (6)	1
3	3/15 mm	BA	13	No	None	Pontine infarction	None	Total occlusion (3)	2
4	3/18 mm	V4 (left)	2	No	None	None	None	Total occlusion (8)	1
5	3/18 mm	V4 (right)	4	No	Thrombus formation	Thrombus formation	3/12 mm 3/15 mm	Total occlusion (5)	4
6	3/18 mm	M3/M2 (right)	0	No	Incomplete opening (distal) Thrombus formation	None	None	Persistent perfusion (8)	5
7	3 × 3/15 mm 2 × 3/9 mm	BA	1	No	None	In-stent-thrombosis Pontine infarction	3/15 mm 3/18 mm	Total occlusion (9)	4
8	3/18 mm	ICA (left)	2	no	None	None	None	–	6

### Retreatment

Three aneurysms underwent repeated treatment with further FD stents before discharge (37.5%). Patient #5 and #7 showed reperfusion and enlargement of the previously treated dissecting aneurysms after vessel recanalization following DAPT. Patient #2 needed retreatment as, following the deployment of a FDS in the right A1/A2 segment, the AcomA aneurysm was still being perfused via the left A1 segment. A second FDS was then deployed into the left A1/A2 segment in order to complete the endovascular treatment before discharge. No hemorrhagic or thromboembolic procedural complications were observed during retreatments.

## Discussion

Flow diversion has significantly expanded the spectrum of intracranial aneurysms treatable by endovascular means. However, published literature on using FD to treat ruptured aneurysms is limited since there appears to be a general sense of reluctance to use DAPT in the presence of an aSAH. A meta-analysis of the use of FDS in ruptured aneurysms has reported a 9% rate of hemorrhagic complications, comprising 5% from aneurysm re-rupture and 4% from other causes (e.g., tracheostomy, EVD, and ventriculoperitoneal shunt placement) [7]. Moreover, there was a 4% rate of symptomatic ischemic complications. Another meta-analysis has recently been performed by Cagnazzo et al. [8], reporting rates of thromboembolic and hemorrhagic complications of 8% and 7%, respectively.

The development of low-thrombogenic neurovascular implants which reduces the extent of antiplatelet therapy (APT) required potentially represents a leap forward in the endovascular treatment of intracranial aneurysms. Devices with lower thrombogenicity would not only be beneficial in treating unruptured aneurysms but even more so for a ruptured aneurysm. Recent advances in polymer coating technology have led to FDS with reduced thrombogenicity becoming available (e.g., PED Shield; Medtronic). Maning et al. [9] used the PED Shield under SAPT to treat 14 patients with aSAH and reported no difference in the rate of acute stent thrombosis, permanent morbidity, or mortality compared with the meta-analysis performed by Cagnazzo et al. [8].

pHPC is a glycan-based hydrophilic multilayer polymer coating which can be applied to nitinol surfaces. It is designed to mimic the biological properties of the glycocalyx, the coverage that can be found on the luminal surface of the endothelium of the arterial wall, making the coated surface hydrophilic and, consequently, less thrombogenic [4]. pHPC has no pharmaceutical effect, is biocompatible with no evidence of acute inflammatory response, and does not interact with the physical properties of the metallic implant [10, 11]. Bhogal et al. [5] recently reported on the use of the p48MW HPC under SAPT in treating five patients with unruptured aneurysms and reported no thromboembolic complications, with just one patient experiencing a minor hemorrhage from the treated aneurysm 2 weeks postoperatively. However, only prasugrel was used for SAPT, and there were no ruptured aneurysms treated in this series.

Our series illustrates the results of using p48MW HPC in ruptured aneurysms under SAPT with either ASA or prasugrel. ASA has the advantage of a low rate of hemorrhagic complications [12]. However, an aSAH activates platelet aggregation [13], which could lead to suboptimal platelet inhibition and explains why increased doses of ASA were required for all but one patient in our series to achieve sufficient platelet inhibition. Alongside this prolonged platelet activation, patients with an aSAH have decreased enteral absorption, which may also lead to the ASA having an insufficient effect [14, 15]. Therefore, we prefer administering ASA intravenously in the acute phase after aSAH. We also assess the dosage with daily Multiplate and VerifyNow tests, adapting the amount as required. Despite these precautions, we observed a 50% rate of intraprocedural thrombus formation, all of them in patients receiving ASA as SAPT. A possible relationship between this thrombus formation and patients not receiving heparin was not observed as all the patients presenting with thrombus had been given an intraprocedural bolus of heparin.

Prasugrel and ticagrelor are P2Y12 platelet receptor antagonists and alternatives to clopidogrel for faster and more efficient platelet inhibition before and after FDS implantation [16]. We have so far not considered using ticagrelor for SAPT due to its short effective period which may result in device thrombosis after skipping just one dose [17]. Prasugrel achieves stronger platelet inhibition with a lower incidence of non-responders compared with clopidogrel [18]. The single complication described in the series by Bhogal et al. was the delayed rupture of a dissecting aneurysm. This complication was believed to be secondary to local degeneration of the arterial wall caused by proteolytic enzymes released by the intra-aneurysmal thrombus and could therefore be considered to be unrelated to prasugrel. In our series, patients who received prasugrel showed less variability and failure in the platelet inhibition before and after the procedure.

### Limitations

Our study has several inherent limitations, which should be considered when interpreting the results. First, this study was limited by its retrospective design and single-center experience with a small sample, which may limit its external validity. Second, because all the aneurysms included were ruptured and in different locations with differing etiologies, patient selection bias is apparent. Third, because not all patients were treated using the same SAPT, and three patients had to be moved from SAPT onto DAPT, the exact effect of HPC was unclear. Physiological conditions and comorbidities of individual patients were not included in this study. However, what we report here is preliminary data, which may help when dealing with similar patients. Further investigations are necessary.

## Conclusions

In this small initial series, the p48MW HPC was used to treat acutely ruptured complex aneurysms under SAPT. Adequate management of the SAPT prior and after treatment remains an issue. Thromboembolic complications are a potential problem that may be more likely to occur under SAPT using ASA rather than prasugrel. Overall, the p48MW HPC FDS appears to be a suitable treatment option for selected ruptured aneurysms. Thromboembolic events are more frequent but clinically less severe than hemorrhage complications, but both of these remain a concern. Large registries and randomized trials addressing safety and efficacy with longer clinical and angiographic follow-up are underway.
